# Nivolumab maintenance in high-risk acute myeloid leukemia patients: a single-arm, open-label, phase II study

**DOI:** 10.1038/s41408-021-00453-z

**Published:** 2021-03-17

**Authors:** Patrick K. Reville, Hagop M. Kantarjian, Farhad Ravandi, Elias Jabbour, Courtney D. DiNardo, Naval Daver, Naveen Pemmaraju, Maro Ohanian, Yesid Alvarado, Lianchun Xiao, Gheath Alatrash, Sanam Loghavi, Caitlin R. Rausch, Gautam Borthakur, Marina Konopleva, Jorge Cortes, Tapan M. Kadia

**Affiliations:** 1 Division of Cancer Medicine, The University of Texas MD Anderson Cancer Center, 1400 Holcombe Boulevard, Houston, TX USA; 2 Department of Leukemia, The University of Texas MD Anderson Cancer Center, 1400 Holcombe Boulevard, Houston, TX USA; 3 Department of Biostatistics, The University of Texas MD Anderson Cancer Center, 1400 Holcombe Boulevard, Houston, TX USA; 4 Department of Stem Cell Transplantation & Cellular Therapy, The University of Texas MD Anderson Cancer Center, 1400 Holcombe Boulevard, Houston, TX USA; 5 Department of Hematopathology, The University of Texas MD Anderson Cancer Center, 1400 Holcombe Boulevard, Houston, TX USA; 6 Division of Pharmacy, The University of Texas MD Anderson Cancer Center, 1400 Holcombe Boulevard, Houston, TX USA; 7 Georgia Cancer Center, Augusta University, 1411 Laney Walker Blvd, Augusta, Georgia

**Keywords:** Immunotherapy, Acute myeloid leukaemia, Phase II trials

Dear Editor,

Patients with high-risk AML ineligible for allogeneic hematopoietic stem cell transplantation (allo-SCT) have poor outcomes and low likelihood of cure^1^. Maintenance cytotoxic chemotherapy in AML has consistently failed to show a benefit^2^. Recent data suggest that there may be a role for hypomethylating agents (HMAs) as maintenance therapy in older individuals with AML^3,4^.

Therapies that engage the immune system have the ability to induce durable remissions. A major mechanism in the maintenance of remission and cure of AML with allo-SCT is graft-versus-leukemia effect^5^. Given the need for novel therapeutic approaches for high-risk AML patients, we designed a pilot phase II clinical trial studying the efficacy and safety of the immune checkpoint inhibitor nivolumab as maintenance therapy in AML. Here we report the results of the first single arm, open-label trial of maintenance nivolumab in patients with high-risk AML in remission not being considered for allo-SCT.

Eligible patients had adequate organ function, an ECOG performance status of ≤2, and AML in remission (defined as CR, CR with incomplete hematologic recovery [CRi], or partial remission [PR]). Patients were defined as having high-risk disease by any of the following: in 1st CR with secondary AML; high-risk cytogenetics at diagnosis; fms-related tyrosine kinase 3 internal tandem duplication mutated at diagnosis; the presence of measurable residual disease assessed by flow cytometry at time of enrollment; or 2nd CR or greater regardless of disease characteristics at the time of initial diagnosis. Patients must have received induction and at least one cycle of consolidation chemotherapy and should have achieved a CR within 12 months of protocol enrollment.

Patients must have not been on steroids (>10 mg prednisone/day or equivalent) or other immunosuppressive medication. Patients with a history of autoimmune disease, positive for hepatitis B surface antigen expression, active hepatitis C infection, or known HIV infection were excluded. The study was approved by the University of Texas MD Anderson Cancer Center Institutional Review Board. All patients signed a written informed consent and the trial was conducted in accordance with the Declaration of Helsinki.

Patients received nivolumab at a dose of 3 mg/kg intravenously every 2 weeks. Cycles repeated every 28 days in the absence of disease progression or unacceptable toxicity. After cycle 6, patients received nivolumab every 4 weeks. After cycle 12, patients received nivolumab every 3 months until disease relapse. All toxicity was graded by Common Terminology Criteria for Adverse Events (CTCAE) version 4.03.

The primary outcome was recurrence-free survival (RFS) rate at six months. Secondary outcomes were to evaluate measurable residual disease (MRD) by flow cytometry as a predictor of response and MRD dynamics with nivolumab therapy; to evaluate time to relapse and overall survival; to evaluate the toxicity profile of nivolumab among patients with AML.

MRD was assessed prior to disease enrollment and as a part of standard practice with each bone marrow biopsy. Multicolor flow cytometry was utilized to assess MRD status to achieve a sensitivity of 0.01%^6^.

For the primary efficacy endpoint, the study was continuously monitoring RFS for futility and the study was to be stopped early if at any time the data suggest that there is less than 25% probability that the median RFS was longer than that in the historical data, 8 months that corresponds to an RFS rate at 6 months of 59.5%. The study was continuously monitored for toxicity and the trial was to be stopped if at any point there was more than an 88% probability that the toxicity rate (defined as any treatment-related clinically significant grade 3 or worse non-hematologic event) of the maintenance therapy with nivolumab is greater than 30%.

The Kaplan–Meier method was used to estimate the median recurrence-free and overall survival probabilities. Statistical analyses were carried out using R. This study is registered with ClinicalTrials.gov, NCT02532231.

From November 11, 2015 through August 15, 2018, 15 patients were enrolled. The median age was 56 (range: 31–71). Based on the European Leukemia Network (ELN) classification, 6 (40%) were adverse, 4 (27%) were intermediate, and 5 (33%) were favorable risk at diagnosis. Nine of the fifteen patients (60%) had detectable MRD at the time of enrollment. At enrollment, 7 patients (47%) were in CR, 7 (47%) patients were CRi, and one patient was in PR. Eight patients were in the first remission and seven were in second remission or greater (Supplemental Table 1).

Patients received a median of 6 (range: 1–23) cycles of therapy. With a median follow up of 30.4 months, the estimated 6-month RFS is 57.1% (95% CI: 36.3–89.9%) and median RFS was 8.48 months (95% CI: 2.14–NE) (Fig. 1A). Two patients proceeded to allo-SCT. One patient, received a peripheral blood stem cell allo-SCT 3 months after last dose of nivolumab, developed grade 4 gastrointestinal graft-versus-host disease and died. The other patient did not develop GvHD. The median overall survival has not yet been reached (95% CI: 10.3 months–NE) (Fig. 1B). Cause of death and post-protocol therapies are outlined in Supplementary Table 2.

**Fig. 1:**
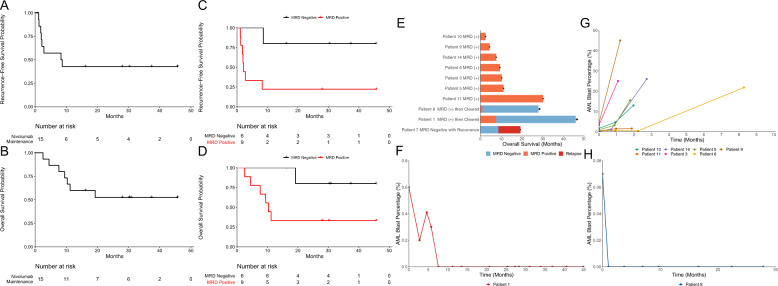
Survival and MRD Dynamics. **A** Kaplan–Meier curve showing recurrence-free survival of the fifteen patients treated with maintenance nivolumab. **B** Kaplan–Meier curve showing overall survival of the fifteen patients treated with maintenance nivolumab. **C** Kaplan–Meier curve showing progression-free survival stratified by MRD status at study enrollment. **D** Kaplan–Meier curve overall survival stratified by MRD status at study enrollment. **E** Swimmer plot showing overall survival of nine patients with detectable MRD at enrollment along with one patient with undetectable MRD that relapsed. Black dot represents patient death while black arrow represents ongoing MRD negative CR. **F** MRD dynamics of the remaining seven patient with positive MRD at enrollment that did not clear MRD while on treatment with maintenance nivolumab. **G** MRD dynamics of patient #1 as measured by flow cytometry while on treatment with maintenance nivolumab. **H** MRD dynamics of patient #8 as measured by flow cytometry while on treatment with maintenance nivolumab.

Six patients were MRD negative at the start of nivolumab maintenance therapy with one experiencing AML recurrence (Fig. 1C/E). Nine patients were MRD positive at the time of enrollment; median level of MRD was 0.6% (range: 0.07–4.3%). Seven of the nine (78%) patients with detectable MRD at enrollment continued to have detectable MRD with progressive disease, despite nivolumab, until frank disease recurrence (Fig. 1G).

Two of nine patients cleared MRD while on treatment with nivolumab. Patient 1 had 0.6% MRD at enrollment and by 7.5 months of treatment with nivolumab had eradication of detectable MRD with no further recurrence (Fig. 1F). This patient had favorable risk disease, with diploid cytogenetics and mutations in *DNMT3A*, *IDH2*, and *NPM1* at diagnosis, and was in MRD positive CR1 at enrollment following induction with cladribine, idarubicin, and cytarabine.

Patient 8 had 0.07% MRD at enrollment and after one month of nivolumab maintenance, no longer had detectable MRD. This patient remains in an MRD negative CR (Fig. 1H). At diagnosis, this patient had intermediate-risk disease, with diploid cytogenetics and a *DNMT3A* mutation, and was in CR2 after induction and re-induction with 7+3.

Adverse events independent of causality are summarized in Table 1. There were 11 grade 3/4 non-immune-related adverse events experienced in 5 patients. 6 patients developed immune-related adverse events (irAE). One patient had grade 2 thyroiditis that required treatment with corticosteroids and thyroid hormone replacement; one patient developed grade 4 alanine aminotransferase elevation and grade 3 aspartate aminotransferase elevation which responded to dose interruption alone; two patients developed grade 3 pneumonitis treated with corticosteroids and dose interruption. These 4 patients resumed treatment with nivolumab after interruption and improvement in irAE. One patient developed warm autoimmune hemolytic anemia as an irAE during cycle 1 and came off study. One other patient discontinued therapy due to irAE, this patient had persistent eosinophilia (grade 1) and allergic rhinitis (grade 2) while on maintenance nivolumab which was discontinued after 13 cycles while in an MRD negative CR and has remained alive and without recurrence since discontinuation. After discontinuation, these symptoms improved.

**Table 1:** Adverse events.

	Grade			
Adverse event, *n* (%)	G1	G2	G3	G4
*Blood and lymphatic system disorders*				
Eosinophilia	1 (7%)			
Febrile neutropenia			1 (7%)	
Hemolysis				1 (7%)
*Cardiac disorders*				
Chest Pain	2 (13%)			
Hypotension			1 (7%)	
*Endocrine disorders*				
Hypothyroidism		1 (7%)		
*Eye disorders*				
Photophobia	1 (7%)			
*Gastrointestinal disorders*				
Abdominal Pain			1 (7%)	
Diarrhea	3 (20%)	2 (13%)		
Distension/bloating, abdominal	1 (7%)			
Gastroesophageal reflux disease		1 (7%)		
Mucositis/stomatitis	1 (7%)			
Nausea		1 (7%)		
Sore Throat		1 (7%)		
Vomiting			1 (7%)	
Oral Pain	1 (7%)			
*General disorders and administration site conditions*				
Edema Limbs	1 (7%)			
Fatigue	1 (7%)	1 (7%)		
Flu like symptoms	1 (7%)	1 (7%)		
*Infections and infestations*				
Upper respiratory infection		2 (13%)		
Lung Infection				
Sepsis			1 (7%)	
*Investigations*				
Alanine aminotransferase increased		2 (13%)	1 (7%)	1 (7%)
Aspartate aminotransferase increased			1 (7%)	
*Musculoskeletal and connective tissue disorders*				
Arthralgia		1 (7%)		
Back Pain		1 (7%)		
*Nervous system disorders*				
Dizziness	2 (13%)			
*Respiratory, thoracic, and mediastinal disorders*				
Pneumonitis			2 (13%)	
Cough		1 (7%)		
Allergic Rhinitis		1 (7%)		
Cough	1 (7%)			
Hemoptysis		1 (7%)		
Nasal Congestion	1 (7%)			
*Skin and subcutaneous tissue disorders*				
Hyperpigmentation	1 (7%)			
Pruritus/itching	4 (27%)	1 (7%)		
Rash	1 (7%)			

This study demonstrated the safety and feasibility of maintenance nivolumab for patients with high-risk AML. It showed a modest effect in eradicating MRD and extending remissions as a single-agent. Notably, the two patients who achieved MRD eradication on study had the lowest positive levels in the cohort. In high-risk AML patients, the relapse rate is high, with a very short disease free survival^7,8^. The most effective post-remission therapy in high-risk AML continues to be allo-SCT which is not universally available^9,10^. Recently, maintenance oral azacitidine was shown to improve both relapse-free and overall survival^11^. The combination of HMA and nivolumab may have additive effects in the maintenance setting. For instance, the combination of nivolumab and azacitidine in the relapsed/refractory AML setting produced a response rate of 33%^12^. However, in an early analysis of durvalumab in combination with azacitadine for frontline treatment did not appear to improve outcomes for older patients with AML^13^.

Grade 3/4 irAEs were observed in 27% of the patients. The irAEs frequently occurred within 8 weeks after nivolumab initiation with all grade 3–4 irAEs were treated with systemic steroids and/or dose interruptions resulting in toxicity resolution and successful re-challenge with nivolumab in all but 1 patient.

In conclusion, nivolumab maintenance produced recurrence-free survival duration similar to historical observation, but encouraging overall survival in high-risk AML patients not being considered for allo-SCT. Multiple clinical trials are ongoing including a randomized trial of PD-1 inhibitor for eradication of MRD in high-risk AML in remission (NCT02275533). While not supporting the use of single-agent nivolumab in this setting, this data provides background and feasibility for incorporating immune checkpoint blockade in combination trials for maintenance therapy in high-risk AML patients.

## Supplementary information

Supplement
