# Supplementary material for: Nivolumab maintenance in high-risk acute myeloid leukemia patients: a single-arm, open-label, phase II study
Source: Blood Cancer J. 2021 Mar 17;11(3):60. doi: 10.1038/s41408-021-00453-z (PMC7969746; doi:10.1038/s41408-021-00453-z)
Supplement: Supplementary file 1 — Supplement [file 41408_2021_453_MOESM1_ESM.docx]

**Supplement**

- 1. **Supplementary Methods**
  2. **Supplementary Table 1**
  3. **Supplementary Table 2**
  4. **Study Protocol**
  5. **Supplementary References**

**Section:**

- 1. **Supplementary Methods**

**Mutational Analysis by Next Generation Sequencing**

We performed mutation analysis using a customized 28-gene next generation sequencing (NGS) panel. Briefly, genomic DNA was extracted from BM aspirates or PB. Amplicon-based NGS targeting of the entire coding regions of a panel of 28 genes associated with myeloid neoplasms was performed using a MiSeq platform (Illumina, San Diego, CA, USA). The genes analyzed were (*ABL1, ASXL1, BRAF, DNMT3A, EGFR, EZH2, FLT3, GATA1, GATA2, HRAS, IDH1, IDH2, IKZF2, JAK2, KIT, KRAS, MDM2, MLL, MPL, MYD88, NOTCH1, NPM1, NRAS, PTPN11, RUNX1, TET2, TP53, and WT1*). Briefly, a sequencing library was prepared using 250 ng of DNA template. Equal quantities of DNA from purified sequencing libraries were used for TruSeq paired-end sequencing on the MiSeq sequencer using the MiSeq Reagent Kit v2 (500 cycles). A minimum quality score of AQ30 is required for a minimum of 75% of bases sequenced ensuring high-quality sequencing results. Variant calling was performed with Illumina MiSeq Reporter Software using human genome build 19 (hg 19) as a reference and reads were aligned using the Integrative Genomics Viewer (IGV, Broad Institute, MA, USA)^1^. For clinical reporting, a minimum sequencing coverage of x250 (bidirectional true paired-end sequencing) was required. The analytical sensitivity was established at 5% mutant reads in a background of wild-type reads. Matched germline DNA was not available for comparison.  *CEBPA* mutations were assessedusing PCR followed by Sanger sequencing. FLT3 (ITD and TKD) and NPM1 mutations were also screened using polymerase chain reaction (PCR) followed by capillary electrophoresis on Genetic Analyzer (Applied Biosystems, Foster City, CA), as described previously^2^.

**Measurable Residual Disease Assessment**

Eight-color flow cytometry analysis was performed using a combination of the following 4 tubes: (1) CD7-FITC, CD33-PE, CD19 PerCP-Cy5.5, CD34-PE-Cy7, CD13-APC, CD38-BV421, CD45-V500; (2) HLADR-FITC, CD117-PE, CD4-PerCP-Cy5.5, CD34-PE-Cy7, CD123-APC, CD19-eF780, CD38-BV421, CD45-V500; (3) HLA-DR-FITC, CD36-PE, CD56-PerCP-Cy5.5, CD34-PE-Cy7, CD64-APC, CD19-eF780, CD14-V450, CD45-V500; and (4) CD5-FITC, CD2-PE, CD22-PerCP-Cy5.5, CD34-PE-Cy7, CD38-APC, CD19-eF780, CD15-V450, CD45-V500. All antibodies were obtained from Becton Dickinson (San Jose, CA) or eBioscience (San Diego, CA). Samples were acquired on FACSCanto II instruments (BD Biosciences, San Diego, CA). MRD was identified in comparison with the known patterns of antigen expression by normal maturing myeloid precursors and monocytes as previously described^3,4^. MRD was quantitated as a percentage of total leukocytes after the exclusion of most red blood cell precursors by forward scatter. At least 200,000 live events were acquired to achieve a minimum sensitivity of 10^−4^ (.01%). MRD was defined as a neoplastic blast population with an abnormal pattern of antigen expression deviating from normal regenerating myeloid progenitors or expressing leukemia associated immunophenotype (LAIP). The abnormal blast population was quantified as a percentage of total events.

- 1. **Supplementary Table 1**

| **Baseline Characteristics (N = 15)** | |
| --- | --- |
| **Characteristic** | **Median [Range] or n (%)** |
| **Age, years** | 56 [31 - 71] |
| **Male, n (%)** | 7 (47) |
| **ECOG performance status, n** |  |
| **0** | 7 (47) |
| **1** | 8 (53) |
| **Disease status, n** |  |
| **CR** | 7 (47) |
| **CRi** | 2 (13) |
| **CRp** | 5 (33) |
| **PR** | 1(7) |
| **MRD status** |  |
| **MRD Positive, n (%)** | 9 (60) |
| **MRD level, %** | 0.6 [0.07 – 4.3] |
| **MRD Negative, n (%)** | 6 (40) |
| **Prior Lines of Therapy, n (%)** |  |
| **1** | 8 (53) |
| **2** | 4 (27) |
| **3** | 2 (20) |
| **4** | 0 (0) |
| **5** | 1 (7) |
| **Hemoglobin, g/dl** | 10.1 [7.8 - 14.9] |
| **Platelets, 10^3^/μl** | 83 [27 - 272] |
| **White blood cell count (WBC), 10^3^/μl** | 3.3 [0.8 - 8.1] |
| **Absolute Neutrophil Count, 10^3^/μl** | 1.7 [0.03 - 5.54] |
| **Peripheral Blood Blast count, %** | 0 [0 - 2] |
| **Bone Marrow Blast count, %** | 1 [0 - 6] |
| **ELN risk, n (%)** |  |
| **Favorable** | 5 (33) |
| **Intermediate** | 4 (27) |
| **Adverse** | 6 (40) |
| **Somatic Mutations, n (%)** |  |
| ***ASXL1*** | 2 (13) |
| ***DNMT3A*** | 5 (33) |
| ***EZH2*** | 1 (7) |
| ***FLT3-ITD*** | 1 (7) |
| ***IDH2*** | 3 (20) |
| ***JAK2*** | 1 (7) |
| ***NPM1*** | 3 (20) |
| ***NRAS*** | 1 (7) |
| ***RUNX1*** | 1 (7) |
| ***TET2*** | 2 (13) |
| ***TP53*** | 3 (20) |
| **None Identified** | 2 (13) |

**1.4 Supplementary Table 1**

| **Summary of individual patient clinical data** | | | | | | | | |
| --- | --- | --- | --- | --- | --- | --- | --- | --- |
| **Patient** | **MRD** | **Treatment Discontinued** | **Reason for Treatment Discontinuation** | **Relapse** | **Died** | **Cause of Death** | **Pre-Protocol/Induction Therapies** | **Post-Protocol Therapies** |
| **1** | MRD POS | N | NA | N | N | NA | CLIA | None |
| **2** | MRD NEG | N | NA | N | N | NA | 7+3, HiDAC | None |
| **3** | MRD POS | Y | Disease progression, relapse | Y | Y | Sepsis due to vancomycin resistant enterococcus bacteremia | 7+3, HiDAC, FLAG, BIDFA | Azacitidine+Lirilumab, IMGN779, BID FA, decitabine |
| **4** | MRD NEG | N | NA | N | N | NA | FAI | None |
| **5** | MRD POS | Y | Disease progression, relapse | Y | Y | AML, Hospice | 7+3, FLAG-Ida | SL-401, decitabine+venetoclax, FAI+decitabine, DS-3032B, CECA |
| **6** | MRD POS | Y | Disease progression, relapse | Y | Y | AML | Sotatercept, 7+3+enasidenib | Decitabine |
| **7** | MRD NEG | Y | Disease progression, relapse | Y | Y | GVHD Complications | 7+3, FIA | decitabine+midostaurin, HSCT |
| **8** | MRD POS | N | NA | N | N | NA | 7+3 twice | None |
| **9** | MRD POS | Y | Disease progression, relapse | Y | Y | AML, Hospice | CECA | Decitabine |
| **10** | MRD POS | Y | Disease progression, relapse | Y | Y | Sepsis due to fusarium infection | CLIA | Venetoclax+low dose AraC |
| **11** | MRD POS | Y | Disease progression, relapse | Y | N | NA | FLAG-Ida+GO | HSCT |
| **12** | MRD NEG | Y | Toxicity/Autoimmune Hemolytic Anemia | N | N | NA | FLAG-Ida+GO | None |
| **13** | MRD NEG | N | NA | N | N | NA | FLAG-Ida+GO | None |
| **14** | MRD POS | Y | Disease progression, relapse | Y | Y | Sepsis due to Candida glabrata candidemia and enterococcus faecium bactermia | 7+3, daunorubicin+ara-C+etoposide, HiDAC | Aza+Nivo+Ipilimumab |
| **15** | MRD NEG | N | NA | N | N | NA | CLIA2+Sorafenib | None |

**1.5 Study Protocol**

**PD-1 Inhibition with Nivolumab for the Treatment of Patients with Acute Myeloid Leukemia in Remission at High Risk for Relapse**

**Department of Leukemia**

**MD Anderson Cancer Center**

# OBJECTIVES

- 1. **Primary Objectives:**

1. To evaluate the anti-leukemic effects of Nivolumab in patients with Acute Myeloid Leukemia (AML) who have achieved a 1^st^ complete remission (CR) after induction chemotherapy and consolidation chemotherapy and have high risk for relapse, or have achieved a 2^nd^ CR. The primary endpoint is defined as recurrence-free survival (RFS) rate at 6 months.

# Secondary Objectives:

1. To evaluate the immunologic responses to Nivolumab among patients with AML in CR status post standard chemotherapy

2. To determine whether response to Nivolumab correlate with immunologic responses.

3. To evaluate assessment of Measurable Residual Disease (MRD) by flow cytometry as a predictor of response to immune therapy in treatment of AML and changes during the course of therapy with nivolumab

4. To evaluate time to relapse and overall survival

5. To evaluate the toxicity profile of Nivolumab among patients with AML in CR

# 2.0 BACKGROUND

**1. Acute Myeloid Leukemia (AML)**: AML is an aggressive hematologic malignancy characterized by a heterogeneous clinical presentation and overall poor prognosis. For over 4 decades, the standard of care for treatment of AML remains a backbone of anthracycline combined with cytarabine-based therapies with little changes in overall survival. The overall survival among elderly patients with AML is particularly dismal. Multiple breakthroughs in diagnostics and better characterization of the disease into risk stratified prognostic groups have been achieved through advances in novel chromosomal and molecular biomarker classification.

**2. Treatment of Relapsed/Refractory AML:** Once AML is found to have relapsed, there are unfortunately very few treatment options available to patients with this devastating disease. If relapse is found to be greater than one year from achievement of CR, high dose cytarabine is often times administered as the standard of care. If relapse is found to be less than 1 year from CR date, this indicates a particularly poor prognosis disease and no standard of care treatment options yet exist for this patient population and recommendations include enrolling patients on available clinical trials if feasible. Even if patients are able to achieve 2^nd^ CR, evidence shows that these patients are at very high risk of relapse of their AML; thus there is a demonstrated need for developing therapies in the relapsed/refractory setting in AML and therapeutic modalities are needed in order to help decrease the risk of relapse.

**3. Treatment of Higher risk AML at completion of standard induction and consolidation:** Current standard of care in AML features induction chemotherapy followed by consolidation strategy (chemotherapy versus stem cell transplantation in selected patient populations). At the conclusion of induction and consolidation, there is yet no standard of care for “maintenance” strategies, especially in those subgroups of AML patients demonstrating a particularly high risk of relapse (e.g. FLT3-ITD mutated patients, high risk cytogenetics (monosomies of either chromosome 5 or 7 or complex cytogenetics), secondary or treatment-related AML, or those in 2^nd^ CR). This subgroup of patients is at very high risk of relapse with no current standard treatment options except active surveillance once therapy for induction and consolidation has been completed. Review of our own experience shows that the RFS of frontline patients with high risk features is similar to any patient with AML who in their second CR (ie. relapsed after frontline therapy), suggesting that these groups can be studied together.

**4. Immune modulation in AML**: Immune-mediated events may participate in the suppression of leukemia, as malignant cells in hematologic malignancies in general are thought to be sensitive to lysis by T cells. The most obvious proof of this effect is the strong graft-versus-leukemia effect in myeloid leukemias in general and in AML in particular. Thus, developing immunologic approaches to treat AML is an attractive therapeutic approach. One approach has used PR1, a nonapetide derived from proteinase 3. Proteinase 3 is a serine protease induced during neutrophil differentiation that is overexpressed in myeloid leukemias.^5^ PR1 is presented through HLA-A2.1^6^ and cytotoxic T lymphocytes (CTL) specific for PR1 inhibit colony formation in an HLA-restricted manner.^5^ These CTL are cytotoxic against fresh myeloid leukemia cells from HLA-A2 patients, demonstrating PR1 specificity of this response.^7^ PR1 is being investigated as a vaccine for patients with myeloid malignancies who have failed prior therapies.^4^ In a recent study, 35 patients with myeloid malignancies were treated. Responses were observed among patients with CML, AML and MDS, and clinical response correlated with immune response in some of these cases.^8^ Scheibenbogen et al reported that T cells recognizing HLA-A2.1-binding epitopes from WT1 or proteinase 3 could be detected ex vivo in patients with acute myeloid leukemia.^9^ In addition, several studies have suggested that cytotoxic effector cells, including T cells and NK cells may play a role in preventing recurrence of AML.^10-13^ The clinical significance of these observations has not been investigated, and there has been little clinical experience with immune-modulating approaches in AML. We conducted a pilot study of Interluekin-2 in patients with AML in first complete remission. A comparison with matched historical controls suggested benefit but the population treated was heterogeneous.^14^ Later, a phase 3 randomized trial treated patients with AML in first or subsequent remission wit interleukin-2 and histamine versus no further therapy after they had completed induction and maintenance. The study showed an improvement in leukemia-free- and overall survival for patients receiving the Interleukin-2 + histamine combination.^15^ Thus, we hypothesize that immune modulation in AML may lead to tumor suppression.

T-cell exhaustion, a state of T-cell dysfunction characterized by impaired killing, hypoproliferation and decreased production of cytokines, has been characterized in solid tumors^16^ and, more recently, in AML animal models.^17^ These lymphocytes are characterized by the co-expression of Tim-3 and PD-1 on CD8+ T-cells that are deficient in their ability to produce a cytokine response in response to PDL1-expressing AML cells. Several studies have suggested that PD-1 blockade can enhance recruitment of effector T-cells and produce therapeutic anti-cancer immunity.^18,19^ Recently, the use of an anti-PD-1 antibody (lambrolizumab) was reported to induce response in 38% of patients with advanced melanoma (52% among those treated at the highest tolerated dose)^20^.

Nivolumab (BMS-936558, MDX-1106) is a fully human immunoglobulin G4 (IgG4) monoclonal anti-PD-1 antibody that, upon binding to PD-01, prevents its interaction with its ligands PDL1 and PDL2. Studies in melanoma have shown evidence of clinical activity with a favorable toxicity profile. Among 90 patients with advanced melanoma treated with nivolumab in a phase 1 study, 25% responded, with responses observed in both ipilimumab-naive and ipilimumab-exposed patients.^21^

In this study we propose to investigate the role of nivolumab in treating patients with AML in CR at high risk for relapse to determine whether treatment with this anti-PD-1 antibody may prevent AML relapse.

**2.4 Anti-PD1 (Nivolumab, BMS-936558, MDX1106):**

**2.4.1 Mechanism of Action**

Immune activation is tightly regulated by co-stimulatory (e.g. CD28 and ICOS) and co-inhibitory (e.g. CTLA-4 and PD-1) receptors expressed on T cells. Agonistic antibodies against co-stimulatory T cell receptors and blocking antibodies against co-inhibitory T cell surface receptors have both been shown to potentiate T cell activation for tumor cell killing.

PD-1 is mainly expressed by activated CD4+ and CD8+ T cells, as well as APCs. It has two ligands, PD-L1 and PD-L2, with distinct expression profiles.^22^ PD-L1 is expressed not only on APCs, but also on non- hematopoietic cells, including tumor cells. Expression of PD-L2 is largely restricted to APCs including macrophages and myeloid dendritic cells, as well as mast cells. The role of PD-1 as a negative regulator of T cells was best demonstrated by the finding that PD-1 deficient mice developed significant autoimmunity with high titers of autoantibodies.^23,24^ Subsequently, blocking antibodies against PD-1 were shown to activate immune responses that resulted in reduction of tumor metastasis and tumor growth in a number of experimental tumor models.^18,25^ Consistent with the immune inhibitory role of PD-1/PD-L1/2 signaling, forced expression of PD-L1 in murine tumor cell lines allowed increased tumor growth in vivo, which was otherwise kept in check by T cells. The inciting effect of PD-L1 on tumor growth was reversed by blocking anti-PD-L1 antibodies.^26^

Nivolumab (BMS-936558) is a fully human, IgG4 (kappa) isotype, monoclonal antibody that binds PD-1. Blockade of the PD-1 pathway by nivolumab was studied using the mixed lymphocyte reaction (MLR). PD-1 blockade resulted in a reproducible enhancement of both proliferation and IFN-γ release in the MLR. The effect of nivolumab on antigen-specific recall response was investigated using a CMV-restimulation assay with human peripheral blood mononuclear cells (PBMCs), and was evaluated by ELISA. These data indicated that nivolumab, versus an isotype-matched control antibody, augmented IFN-γ secretion from CMV-specific memory T cells in a dose-dependent manner. PD-1 blockade by nivolumab is therefore considered a promising immunotherapeutic strategy.

**2.4.2 Summary of Safety Results from Nivolumab Program**

For a complete review of clinical information, please refer to the nivolumab Investigator Brochure.

**2.4.2.1 Summary of Safety**

The monitoring of subject safety during and after a clinical study with nivolumab, including any special monitoring precautions, tests, or observations, and the proper means of recording and reporting adverse safety information (ie, AEs and abnormal laboratory values) will follow the procedures outlined in the specific study protocol.

The overall safety experience with nivolumab is based on experience in approximately 1500 subjects as either a monotherapy or in combination with other therapeutics. In general for monotherapy, the safety profile is similar across tumor types. The one exception is pulmonary inflammation AEs which may be numerically greater in subjects with NSCLC possibly because in some cases it can be difficult to distinguish between nivolumab-related and unrelated causes of pulmonary symptoms and radiographic changes. The most frequently reported treatment- related AE is fatigue which is almost always low grade.

The safety profile is generally consistent across completed and ongoing clinical trials with no MTD reached at any dose tested up to 10 mg/kg. There was no pattern in the incidence, severity, or causality of AEs to nivolumab dose level. Most related AEs are thought to be due to the effects of inflammatory cells on specific tissues. Most AEs were low-grade (Grade 1 to Grade 2) with relatively few related high-grade (Grade 3 to Grade 4) AEs. Most high-grade events were manageable with use of corticosteroids or hormone replacement therapy (endocrinopathies).

2.4.2.2 Clinical Safety in Advanced Malignancies (Nivolumab Monotherapy)

A total of 306 subjects with treatment-refractory malignancies have been treated in an ongoing, Phase 1 multidose study (MDX1106-03, CA209003). This is an ongoing phase I dose-escalation study of nivolumab monotherapy in patients with advanced cancers; 1, 3, or 10 mg/kg nivolumab and 0.1 and 0.3 mg/kg (included as part of Amendment 4) administered by IV Q2W; treatment up to 2 years. Results were published by Topalian et al. (NEJM 2012).^27^ The baseline disease diagnosis by treatment for MDX1106-03 is provided in Table 2.4.2.2-1. A review of the safety data by tumor type (RCC, NSCLC, mCRPC, CRC, and melanoma) did not show any clinically meaningful differences in the proportion of subjects with AEs noted across tumor type.

Table 2.4.2.2-1: Baseline Disease Diagnosis by Treatment - MDX1106-03

**No. of Subjects**

| **Nivolumab (mg/kg)** | **0.1 mg/kg** | **0.3 mg/kg** | **1 mg/kg** | **3 mg/kg** | **10 mg/kg** | **TOTAL** |
| --- | --- | --- | --- | --- | --- | --- |
| **MDX1106-03,**  **Total N** | **17** | **18** | **86** | **54** | **131** | **306** |
| NSCLC | 0 | 0 | 33 | 37 | 58 | 128 |
| Melanoma | 17 | 18 | 35 | 17 | 20 | 107 |
| RCC | 0 | 0 | 18 | 0 | 16 | 34 |
| mCRPC | 0 | 0 | 0 | 0 | 17 | 17 |
| CRC | 0 | 0 | 0 | 0 | 19 | 19 |

Abbreviations: CRC: colorectal adenocarcinoma; mCRPC: metastatic castration-resistant prostate cancer; NSCLC: non-small cell lung cancer; RCC: renal cell carcinoma;

Source: Preliminary data, CA209003. Clinical data cut-off date 18-Mar-2013.

2.4.2.3 Adverse Events

There was no pattern in the incidence, severity, or causality of AEs related to the dose of nivolumab, between 1 and 10 mg/kg, in MDX1106-03. Of the 306 treated subjects in MDX1106-03, 303 (99.0%) subjects have at least 1 reported AE regardless of causality (Table 2.4.2.3-1). The most frequently reported AEs were fatigue (54.9%), decreased appetite (35.0%), diarrhea (34.3%), nausea (30.1%), and cough (29.4%). Treatment-related AEs were reported in 230 (75.2%) of the 306 subjects. The most frequently reported treatment-related AEs were fatigue (28.1%), rash (14.7%), diarrhea (13.4%), and pruritus (10.5%). Most treatment-related AEs were low grade. **Treatment-related Grade 3-4 AEs were reported in 52 (17.0%) of subjects.** The most frequently reported treatment-related high grade AE was fatigue (6.5%).

Table 2.4.2.3-1: Summary of Adverse Events Reported in ≥15% of All Treated Subjects - MDX1106-03

**No. of Subjects (%)**

**Preferred Term AEs regardless of causality Treatment-related AEs**

**Any Grade N=306**

**Grade 3-4 N=306**

**Any Grade N=306**

**Grade 3-4 N=306**

**Any AE 303 (99) 127 (42) 230 (75) 52 (17)**

Fatigue 168 (55) 20 (7) 86 (28) 7 (2)

Decreased appetite 107 (35) 3 (1) 28 (9) 1(0.3)

Diarrhea 105 (34) 3 (1) 41 (13) 3 (1)

Nausea 92 (30) 9 (3) 27 (9) 2 (1)

Cough 90 (29) 4 (1) 11 (4) 1 (0.3)

Dyspnea 80 (26) 27 (9) 11 (4) 0

Constipation 78 (26) 2 (1) 5 (2) 0

Rash 74 (24) 0 45 (15) 0

Vomiting 70 (23) 7 (2) 10 (3) 1 (0.3)

Back pain 68 (22) 7 (2) 3 (1) 1 (0.3)

Arthralgia 63 (21) 4 (1) 15 (5) 0

Pyrexia 61 (20) 1 (0.3) 17 (6) 0

Headache 59 (19) 1 (0.3) 8 (3) 0

Oedema peripheral 59 (19) 1 (0.3) 3 (1) 0

Dizziness 56 (18) 1 (0.3) 10 (3) 0

Pruritus 56 (18) 1 (0.3) 32 (11) 1 (0.3)

Weight decreased 48 (16) 1 (0.3) 11 (4) 0

Malignant neoplasm progression

48 (16) 4 (1) 1 (0.3) 1 (0.3)

Abbreviations: AE: adverse event

Source: Preliminary data, MDX1106-03. Clinical data cut-off date: 18-Mar-2013

2.4.2.4 Select Adverse Events

Select AE categories (events with a potential inflammatory mechanism requiring more frequent monitoring and/or unique intervention such as immunosuppressants and/or endocrine replacement therapy) include: GI AEs, pulmonary AEs, renal AEs, hepatic AEs, skin AEs, and endocrinopathies. In addition, Select AEs include a category for infusion reactions. Each category is composed of a discrete set of preferred terms, including those of greatest clinical relevance. These Select AEs are considered events of interest based on the mechanism of action and were previously referred to as immune-related AEs or immune-mediated AEs.

The frequencies of these events are summarized in Table 2.4.2.4-1. The 10 mg/kg cohort had numerically greater frequency of high-grade select AEs including the subcategories of endocrinopathies, GI, pulmonary, and infusion reactions.

Table 2.4.2.4-1: Treatment-related Select Adverse Events by Treatment - All CTC Grades Reported in at Least 10 Treated Subjects in MDX1106-03

| **Preferred Term** | **0.1 mg/kg n=17**  Any Grade  Grade 3-4 | | **0.3 mg/kg n=18**  Any Grade  Grade 3-4 | | **1 mg/kg n=86**  Any Grade  Grade 3-4 | | **3 mg/kg n=54**  Any Grade  Grade 3-4 | | **10 mg/kg n=131**  Any Grade  Grade 3-4 | | **Total N=306**  Any Grade  Grade 3-4 | |
| --- | --- | --- | --- | --- | --- | --- | --- | --- | --- | --- | --- | --- |
| **Any Select AE** | **8 (47)** | **1 (5.9)** | **9 (50)** | **0** | **42 (49)** | **3 (4)** | **23 (43)** | **2 (4)** | **58 (44)** | **13 (10)** | **140 (46)** | **19 (6)** |
| **Any Endocrinopathies** | **4 (24)** | **0** | **2 (11)** | **0** | **9 (11)** | **0** | **4 (7)** | **0** | **10 (8)** | **3 (2)** | **29 (10)** | **3 (1)** |
| Endocrinopathies Thyroid | 3 (18) | 0 | 2 (11) | 0 | 9 (11) | 0 | 4 (7) | 0 | 8 (6) | 2 (2) | 26 (9) | 2 (1) |
| Blood TSH increased | 2 (12) | 0 | 1 (6) | 0 | 2 (2) | 0 | 2 (4) | 0 | 4 (3) | 1 (1) | 11 (4) | 1 (0.3) |
| Hypothyroidism | 1 (6) | 0 | 1 (6) | 0 | 5 (6) | 0 | 1 (2) | 0 | 3 (2) | 1 (1) | 11 (4) | 1 (0.3) |
| **Any Skin AEs** | **3 (18)** | **0** | **5 (28)** | **0** | **27 (31)** | **0** | **12 (22)** | **0** | **28 (21)** | **1 (1)** | **75 (25)** | **1 (0.3)** |
| Rash | 3 (18) | 0 | 3 (17) | 0 | 20 (23) | 0 | 5 (9) | 0 | 14 (11) | 0 | 45 (15) | 0 |
| Pruritus | 0 | 0 | 1 (6) | 0 | 15 (17) | 0 | 3 (6) | 0 | 13 (10) | 1 (1) | 32 (11) | 1 (0.3) |
| **Any GI AE** | **1 (6)** | **0** | **2 (11)** | **0** | **19 (22)** | **0** | **7 (13)** | **0** | **14 (11)** | **3 (2)** | **43 (14)** | **3 (1)** |
| Diarrhea | 1 (6) | 0 | 2 (11) | 0 | 19 (22) | 0 | 6 (11) | 0 | 13 (10) | 3 (2) | 41 (13) | 3 (1) |
| **Any hepatic AE** | **0** | **0** | **2 (11)** | **0** | **8 (9)** | **0** | **3 (6)** | **2 (4)** | **5 (4)** | **2 (2)** | **18 (6)** | **4 (1)** |
| ALT increased | 0 | 0 | 1 (6) | 0 | 6 (7) | 0 | 1 (2) | 0 | 3 (2) | **1 (1)** | 11 (4) | 1 (0.3) |
| **Any Pulmonary AE** | 1 (6) | **0** | 1 (6) | **0** | **6 (7)** | **3 (4)** | **2 (4)** | **0** | **7 (5)** | **3 (2)** | **17 (6)** | **6 (2)** |
| Pneumonitis | 1 (6) | 0 | 0 | 0 | 4 (5) | 2 (2) | 1 (2) | 0 | 6 (5) | 2 (2) | 12 (4) | 4 (1) |
| **Infusion reaction** | **0** | **0** | **1 (6)** | **0** | **3 (4)** | **0** | **3 (6)** | **0** | **8 (6)** | **2 (2)** | **15 (5)** | **2 (1)** |
| Infusion-related reaction | 0 | 0 | 1 (6) | 0 | 3 (4) | 0 | 3 (6) | 0 | 5 (4) | 0 | 12 (4) | 0 |
| Hypersensitivity | 0 | 0 | 0 | 0 | 0 | 0 | 1 (1.9) | 0 | 3 (2) | 2 (2) | 4 (1) | 2 (1) |

Abbreviations: ALT: alanine aminotransferase, TSH: thyroid stimulating hormone

Source: Preliminary data, MDX1106-03. Clinical data cut-off date: 18-Mar-2013

2.4.2.5 Adverse Events Leading to Discontinuation

At least 1 treatment-related AE leading to discontinuation was reported in 32 (10.5%) of the 306 treated subjects. Grade 3-4 treatment-related events were reported in 14 (4.6%) subjects. The frequency of treatment-related AEs leading to discontinuation was not associated with the dose of nivolumab. Pneumonitis was the most common treatment-related AE leading to discontinuation (8 subjects, 2.6%); pneumonitis reported in 3 (1.0%) subjects was Grade 3-4. Treatment-related AEs reported in at least 2 subjects included pneumonitis (8 subjects, 2.6%), colitis (3 subjects, 1.0%) and myalgia, hepatitis, hypersensitivity, and infusion-related reactions (each reported in 2 subjects, 0.7%). One event of Grade 5 sepsis was reported for 1 subject, a 62-year-old male treated with 1 mg/kg nivolumab.

2.4.2.6 Deaths

As of 18-Mar-2013, 195 deaths have been reported in MDX1106-03 during the course of the study or within 30 days of last dose of study drug. The majority of the deaths were considered secondary to disease progression and malignant disease.

Three subjects in MDX1106-03 died after developing pneumonitis. A 62-year-old male (MDX1106-03-1-699) with NSCLC (adenocarcinoma) in the 1 mg/kg treatment group and a 59-year old male (MDX1106-03-1-3582) with CRC in the 10 mg/kg treatment group both died due to Grade 5 sepsis after having developed Grade 4 pneumonitis. Sepsis and pneumonitis were considered related to study drug by the investigator in both of these cases. In addition, a 40-year- old female (MDX1106-03-5-710) with NSCLC (adenocarcinoma) in the 1 mg/kg treatment group died due to respiratory failure after having developed Grade 4 pneumonitis and tumor progression. In this case, respiratory failure and pneumonitis were considered related to study drug by the investigator.

Additional deaths reported as due to “other” in MDX1106-03 included:

- MDX1106-03-2-3436 (10 mg/kg): Ischemic cardiomyopathy
- MDX1106-03-4-3484 (10 mg/kg): Death due to abdominal pain caused by superior mesenteric vein thrombosis
- MDX1106-03-3-674 (1 mg/kg): Progressive lung cancer

**2.4.3 Summary of Efficacy (Nivolumab monotherapy)**

The clinical activity data presented below are from MDX1106-03 (nivolumab monotherapy).

Table 2.4.3-1: Objective Response Rate and Progression Free Survival 24 Weeks Rate in Melanoma Subjects - MDX1106-03

| **Dose (mg/kg)** | **N** | **ORR** | **PFSR at 24 weeks (%)** |
| --- | --- | --- | --- |
| **All Melanoma** | **107** | **33 (31)** | **44** |
| 0.1 | 17 | 6 (35) | 39 |
| 0.3 | 18 | 5 (28) | 33 |
| 1.0 | 35 | 11 (31) | 51 |
| 3.0 | 17 | 7 (41) | 55 |
| 10.0 | 20 | 4 (20) | 35 |

Abbreviations: ORR: objective response rate, PFSR: progression-free survival rate

Source: Preliminary data, MDX1106-03. Clinical cut-off date: 18-Mar-2013.

Table 2.4.3-2: Objective Response Rate per RECIST 1.0 and Progression Free Survival 24 Weeks Rate by Histology in Non-small Cell Lung Cancer Subjects - MDX1106-03

| **Dose (mg/kg)** | **N** | **Histology** | **ORR**  **No. of Subjects (%)** | **PFSR at 24**  **weeks (%)** |
| --- | --- | --- | --- | --- |
| **All NSCLC** | **129** | **NA** | **22 (17)** | **34** |
| 1.0 | 15 | SQ | 0 | 36 |
|  | 18 | NSQ | 1 (6) | 19 |
| 3.0 | 18 | SQ | 4 (22) | 45 |
|  | 19 | NSQ | 5 (26) | 42 |
| 10.0 | 21 | SQ | 5 (24) | 45 |
|  | 37 | NSQ | 7 (19) | 25 |

Abbreviations: NA: not applicable, NSCLC: non-small cell lung cancer; NSQ: non-squamous, ORR: objective response rate; PFSR: progression free survival rate, SQ: squamous

Source: Preliminary data, MDX1106-03. Clinical cut-off date 18- Mar-2013.

**2.5 BMS 3 mg/kg Dose Rationale and Schedule:**

**N.B. - the 3 mg/kg dose is being carried forward in all hematologic malignancy trials including with dasatinib in advanced CML, NHL, and HL and is the program standard in both solid tumors and hematologic malignancies. The rationale is based upon PD results in patients with solid tumors as follows:**

- - 1. Rationale for nivolumab dose and schedule in NSCLC

The dose and schedule of nivolumab in this study will be 3 mg/kg intravenously (IV) every 2 weeks, based upon a February 24, 2012 analysis of safety, efficacy, and exposure-response data from the ongoing Phase 1 study CA209003. Anti-tumor activity was observed in NSCLC subjects at dose levels of 1, 3 and 10 mg/kg every 2 weeks. Anti-tumor activity appeared to approach a plateau at dose levels of 3 mg/kg and above. Consistent with these observations, the results of exposure‑response analyses showed that the probability of a tumor response tended to approach a plateau for trough concentrations produced by 3 and 10 mg/kg administered every 2 weeks. Nivolumab was adequately tolerated up to 10 mg/kg, the highest dose level tested, and no maximum tolerated dose (MTD) was identified. While the spectrum, frequency, and severity of -related AEs were generally similar across the dose levels tested, the 10 mg/kg dose level had numerically higher rates of Grade 3/4 drug-related SAEs and AEs leading to discontinuation. Based on these observations, a dose of 3 mg/kg IV every 2 weeks was chosen for further study.

- - 1. Nivolumab Dose Selection in Melanoma

Preclinical, PK, and Phase 1 clinical data supported 3 mg/kg IV Q2W as the nivolumab monotherapy dose and schedule selected for further development across all tumor types including melanoma. Specific clinical findings among the 306 subjects treated in MDX1106-03 that contributed to the selection of the Phase 2/3 dose were as follows

- Receptor occupancy was maintained at high levels across the dose range 0.1 to 10 mg/kg and over multiple cycles of drug administration.
- In exposure-response analysis, the probability of response in melanoma, lung, and RCC subjects increased over the range of steady-state trough concentrations observed, but appeared to plateau at the ≥3 mg/kg Q2W dose.
- There was a greater percent of objective responses observed in NSCLC subjects treated with 3 mg/kg (24.3%) and 10 mg/kg (20.3%) nivolumab than with 1 mg/kg (3%) nivolumab.
- The nature, frequency, and severity of adverse events (AEs) were similar across the dose range 0.1 to 10 mg/kg and across tumor types.

The selected Phase 2/3 dose and schedule of nivolumab monotherapy was further evaluated in CA209037. Integrated assessment of data from studies MDX1106-03, CA209037, and CA209066 indicate that 3 mg/kg IV Q2W nivolumab offers an acceptable benefit-risk in this population, based on the consistency of ORR, DOR, other efficacy endpoints, and the safety profile.

**2.6 Rationale for the proposed study**

Despite the high rate of remission in AML, most patients relapse. Continuation of chemotherapy does not only expose the patient to increasing risks for adverse events but is also ineffective. The only approach for maintenance in AML that has been demonstrated to be beneficial in a randomized trial is immunotherapy with interleukin-2 with histamine. We hypothesize that by using nivolumab as maintenance therapy we will induce an immune response that will be able to reduce the risk of relapse in patients at high risk. We thus propose to investigate the role of nivolumab in treating patients with AML in CR at high risk for relapse to determine whether treatment with this anti-PD-1 antibody may prevent AML relapse. If this study suggests a decreased rate of relapse and adequate safety, we would proceed then to a randomized trial to confirm the observation with focus on specific subsets as individual categories (e.g., FLT3 mutated, secondary leukemias, etc).

# 3.0 Study Population

**3.1 Inclusion Criteria**

1. Patients with AML in remission (defined as CR, CR with incomplete platelet recovery –CRp-, CR with incomplete hematologic recovery -CRi-, or partial remission defined as a bone marrow with <10% blasts after therapy with or without hematologic recovery).
2. High risk for relapse defined as:
   1. 1^st^ CR with high risk features for relapse (including history of prior malignancy treated with chemotherapy or radiotherapy, or history of myelodysplastic syndrome, myeloproliferative disorder, chronic myelomonocytic leukemia, MDS/MPN or other hematologic malignancy thought to have evolved to AML [i.e., secondary AML, sAML]; high risk cytogenetics at diagnosis; FLT3 mutated at diagnosis; or presence or measurable residual disease assessed by PCR, cytogenetics, and/or flow cytometry at time of enrollment)
   2. 2^nd^ CR regardless of disease characteristics at the time of diagnosis.
3. Have received induction chemotherapy and at least one cycle of consolidation chemotherapy. Patients should have achieved a CR within 12 months of enrollment onto protocol.
4. No further chemotherapy or SCT planned at the time of enrollment.
5. Age 18 years or older.
6. Adequate organ function: creatinine ≤1.5 x ULN; serum bilirubin ≤1.5 x ULN; AST and ALT ≤2.5 x ULN.
7. ECOG performance status ≤2
8. Females of childbearing potential must have a negative serum or urine beta human chorionic gonadotrophin (β-hCG) pregnancy test result within 24 hours prior to the first dose of treatment and must agree to use an effective contraception method during the study and for 23 weeks after the last dose of the study drug. Females of non- childbearing potential are those who are postmenopausal greater than 1 year or who have had a bilateral tubal ligation or hysterectomy.
9. Males who have partners of childbearing potential must agree to use an effective contraceptive method during the study and for 31 weeks following the last dose of study drug
10. Patients or their legally authorized representative must provide written informed consent.

**3.2 Exclusion Criteria**

1. History of another primary invasive malignancy that has not been definitively treated or in remission for at least 2 years. Patients with non-melanoma skin cancers or with carcinomas in situ are eligible regardless of the time from diagnosis (including concomitant diagnoses).

2. Any major surgery, radiotherapy, chemotherapy, biologic therapy, immunotherapy, experimental therapy within 2 weeks prior to the first dose of the study drugs.

3. Patients with any other known concurrent severe and/or uncontrolled medical condition (e.g. uncontrolled diabetes; cardiovascular disease including congestive heart failure NYHA Class III or IV, myocardial infarction within 6 months, and poorly controlled hypertension; chronic renal failure; or active uncontrolled infection) which, in the opinion of the investigator could compromise participation in the study.

4. Patients unwilling or unable to comply with the protocol.

5. Patients who are on steroids (> 10 mg/day or equivalent) or immune suppression medications.

6. Patients with autoimmune diseases (e.g., rheumatoid arthritis, systemic progressive sclerosis [scleroderma], Systemic Lupus Erythematosus, autoimmune vasculitis [e.g., Wegener’s Granulomatosis]).

7. Patients with a history of Inflammatory Bowel Disease such as Crohn’s disease and ulcerative colitis

8 Patients known to be positive for hepatitis B surface antigen expression or with active hepatitis C infection (positive by polymerase chain reaction or on antiviral therapy for hepatitis C within the last 6 months), or with known HIV infection.

9. Current therapy with other systemic anti-neoplastic or anti-neoplastic investigational agents.

10. Females who are pregnant or lactating

11. Patients with history of previous immunomodulatory therapy.(not including lenalidomide or thalidomide).

# 4.0 TREATMENT PLAN

# 4.1 Study Design

# This is a phase II open label single-arm study to evaluate the efficacy and safety of nivolumab in patients with AML in complete remission but at high risk for relapse. All patients will be registered in CORe.

4.2 Treatment Plan: Patients will receive nivolumab according to the following treatment plan. Individual minor variations in the initiation of therapy, are acceptable as indicated by patient condition and physician judgment. These variations should be documented in the patient’s medical record.

4.2.1 Therapy: Patient will receive Nivolumab 3 mg/kg IV every 2 weeks for 6 cycles. One cycle will be defined as 4 weeks (i.e., 2 planned doses of nivolumab).

4.2.2 Continuation beyond 6 cycles will be allowed for patients deriving benefit without unacceptable toxicity. The dose after 6 cycles may be modified to an every 4 weeks schedule until 12 months from start of therapy, then every 3 months until disease progression.

4.2.3 Patients who discontinue treatment while still in remission or decrease the frequency of administration as described above may have re-treatment at up to every 2 weeks schedule if there is evidence of recurrence of MRD. However, patients with confirmed clinically significant hematologic relapse must be removed from the study.

4.2.4 Treatment may continue until the end of therapy as described in section 4.2.2 or until one of the following criteria applies:

1. Clinically significant progressive disease, or
2. Intercurrent illness that prevents further administration of treatment, or
3. Patient request or
4. General or specific changes in the patient's condition render the patient unacceptable for further treatment in the judgment of the investigator, or
5. Unacceptable toxicity that in the opinion of the investigator makes it unsafe to continue therapy.

**5.0 Study Medications**

**5.1 Nivolumab (Anti-PD1)**

Nivolumab is a fully human, IgG4 (kappa) isotype, monoclonal antibody that binds PD-1. Nivolumab will be supplied in vials of 100 mg (10 mg/mL) and packaged in an open-label fashion. Ten nivolumab vials (each 10 mL) will be packaged within a carton. The vials are not subject specific although there will be specific vial assignments by subject distributed by the Pharmacy in order to track drug usage and re-supply.

**PRODUCT INFORMATION TABLE**: Please also see Drug Information Appendix A

| **Product Description:(Other names = MDX-1106, ONO-4538, anti-PD-1** | | | | | |
| --- | --- | --- | --- | --- | --- |
| **Product Description and Dosage Form** | **Potency** | **Primary Packaging (Volume)/ Label Type** | **Secondary Packaging (Qty) /Label Type** | **Appearance** | **Storage Conditions (per label)** |
| Nivolumab (BMS-936558-01)* Injection drug product is a sterile, non-pyrogenic, single-use, isotonic aqueous solution formulated at 10 mg/mL | 100 mg/Vial (10 mg/mL). | Carton of 5 or 10 vials | 10-cc Type 1 flint glass vials stoppered with butyl stoppers and sealed with aluminum seals. | Clear to opalescent, colorless to pale yellow liquid. May contain particles | **BMS-936558-01 Injection** must be stored at 2 to 8 degrees C (36 to 46 degrees F) and protected from light and freezing |

*Nivolumab may be labeled as BMS-936558-01 Solution for Injection

**5.1.1 Dose Calculation of nivolumab**

Total dose should be calculated as in the following example:

Subject’s actual body weight in kg x 3 mg = total dose in mg

Therefore, a subject weighing 70 kg who is to receive a dose of 3 mg/kg would be administered 210 mg of nivolumab (70 kg x 3 mg/kg = 210 mg).

**5.1.2 Preparation and Dispensing of Nivolumab**

The product storage manager should ensure that the study drug is stored in accordance with the environmental conditions (temperature, light, and humidity) as determined by the Investigator Brochure. If concerns regarding the quality or appearance of the study drug arise, do not dispense the study drug and contact Bristol Myers Squibb immediately.

Investigational product documentation must be maintained that includes all processes required to ensure drug is accurately administered. This includes documentation of drug storage, administration and, as applicable, storage temperatures, reconstitution, and use of required processes (e.g. required diluents, administration sets).

Nivolumab vials must be stored at a temperature of 2°C to 8°C and should be protected from light. If stored in a glass front refrigerator, vials should be stored in the carton. Recommended safety measures for preparation and handling of nivolumab include laboratory coats and gloves.

For details on prepared drug storage and use time of nivolumab under room temperature/light and refrigeration, please refer to the Investigator Brochure section for “Recommended Storage and Use Conditions”. Care must be taken to assure sterility of the prepared solution as the product does not contain any anti-microbial preservative or bacteriostatic agent. No incompatibilities between nivolumab and polyolefin bags have been observed.

Nivolumab is to be administered as an approximately 60 minute IV infusion, using a volumetric pump with a 0.2/0.22 micron in-line filter at the protocol-specified dose. The drug can be diluted with 0.9% normal saline for delivery but the total drug concentration of the solution cannot be below 1 mg/dl. It is not to be administered as an IV push or bolus injection. At the end of the infusion, flush the line with a sufficient quantity of normal saline.

**5.1.3 Administration of nivolumab**

Patients will receive nivolumab as a 60 minute IV infusion on Day 1 and 15 (+/- 3days) of a 28 day treatment cycle. Patients may be dosed no less than 12 days from the previous dosing of nivolumab. Dosing calculations should be based on the actual body weight. If the patient’s weight on the day of dosing differs by > 10% from the weight used to calculate the original dose, the dose must be recalculated. All doses should be rounded to the nearest milligram. There will be no dose modifications allowed.

**5.1.4 Patient Monitoring During Infusion**

Patient vital signs should be monitored prior to dosing, about 15 minutes after initiation of the infusion (then every 15-20 minutes as indicated), at 60 and 120

minutes after completion of the infusion, or longer if indicated, until the vital signs normalize or return to baseline. For subsequent infusions, vital signs should be collected prior to dosing, every 30 minutes during dosing, and 1 hour post dosing.

**5.1.5 Treatment of nivolumab Related Infusion Reactions**

Since nivolumab contains only human immunoglobulin protein sequences, it is unlikely to be immunogenic and induce infusion or hypersensitivity reactions. However, if such a reaction were to occur, it might manifest with fever, chills, rigors, headache, rash, pruritus, arthralgias, hypo- or hypertension, bronchospasm, or other symptoms. All Grade 3 or 4 infusion reactions should be reported within 24 hours to BMS. Grade 3 or 4 infusion reactions will also be reported as an SAE if criteria are met as defined in section 8.4. Infusion reactions should be graded according to NCI CTCAE (version 4.0) guidelines.

Treatment recommendations for nivolumab related infusion reactions are provided below and may be modified based on MD Anderson treatment standards and guidelines, as appropriate:

**For Grade 1 symptoms** (Mild reaction; infusion interruption not indicated; intervention not indicated):Remain at bedside and monitor subject until recovery from symptoms. The following prophylactic premedications are recommended for future infusions: diphenhydramine 50 mg (or equivalent) and/or acetaminophen 325 to 1000 mg at least 30 minutes before additional nivolumab administrations.

**For Grade 2 symptoms** (Moderate reaction requires therapy or infusion interruption but responds promptly to symptomatic treatment [e.g. antihistamines, non-steroidal anti-inflammatory drugs, narcotics, corticosteroids, bronchodilators, IV fluids]; prophylactic medications indicated for ≤ 24 hours):

Stop the nivolumab infusion, begin an IV infusion of normal saline, and treat the subject with diphenhydramine 50 mg IV (or equivalent) and/or acetaminophen 325 to 1000 mg; remain at bedside and monitor subject until resolution of symptoms. Corticosteroid or bronchodilator therapy may also be administered as appropriate. If the infusion is interrupted, then restart the infusion at 50% of the original infusion rate when symptoms resolve; if no further complications ensue after 30 minutes, the rate may be increased to 100% of the original infusion rate. Monitor subject closely. If symptoms recur then no further nivolumab will be administered at that visit. Administer diphenhydramine 50 mg IV, and remain at bedside and monitor the subject until resolution of symptoms. The amount of study drug infused must be recorded on the case report form (CRF). The following prophylactic premedications are recommended for future infusions: diphenhydramine 50 mg (or equivalent) and/or acetaminophen 325 to 1000 mg should be administered at least 30 minutes before additional nivolumab administrations. If necessary, corticosteroids (recommended dose: up to 25 mg of IV hydrocortisone or equivalent) may be used.

**For Grade 3 or Grade 4 symptoms** [Severe reaction, Grade 3: prolonged (i.e. not rapidly responsive to symptomatic medication and/or brief interruption of infusion); recurrence of symptoms following initial improvement; hospitalization indicated for other clinical sequelae (e.g. renal impairment, pulmonary infiltrates), Grade 4: life- threatening; pressor or ventilatory support indicated]:

Immediately discontinue infusion of nivolumab. Begin an IV infusion of normal saline, and treat the subject as follows: Recommend bronchodilators, epinephrine 0.2 to 1 mg of a 1:1,000 solution for subcutaneous administration or 0.1 to 0.25 mg of a 1:10,000 solution injected slowly for IV administration, and/or diphenhydramine 50 mg IV with methylprednisolone 100 mg IV (or equivalent), as needed. Subject should be monitored until the investigator is comfortable that the symptoms will not recur. Nivolumab will be permanently discontinued. Institutional guidelines will be followed for the treatment of anaphylaxis. Remain at bedside and monitor subject until recovery from symptoms. In the case of late-occurring hypersensitivity symptoms (e.g. appearance of a localized or generalized pruritus within 1 week after treatment), symptomatic treatment may be given (e.g. oral antihistamine or corticosteroids).

**5.1.6 Dose Modification**

Dose reductions or dose escalations are not permitted.

**5.2 Dose Delay Criteria:**

Because of the potential for clinically meaningful nivolumab-related AEs requiring early recognition and prompt intervention, management algorithms have been developed for suspected AEs of selected categories.

Dose delay criteria apply for all drug-related adverse events

Nivolumab administration should be delayed for the following:

- Any Grade ≥ 2 non-skin, drug-related AE, with the following exceptions:
  - Grade 2 drug-related fatigue or laboratory abnormalities do not require a treatment delay, However, dose delays should occur for the following laboratory abnormalities:
    - G2 Creatinine, or
    - G2 ALT, AST or total bilirubin
- Any Grade 3 skin, drug-related AE
- Any Grade 3 drug-related laboratory abnormality, with the following exceptions for lymphopenia, leukopenia, AST, ALT, total bilirubin, or asymptomatic amylase or lipase:
  - Grade 3 lymphopenia or leukopenia does not require dose delay.
  - Grade 3 AST, ALT or total bilirubin requires permanent discontinuation of nivolumab
- Any AE, laboratory abnormality, or intercurrent illness which, in the judgment of the investigator, warrants delaying the dose of study medication.

**5.3 Criteria to Resume Treatment:**

Subjects may resume treatment with study drug when the drug-related AE(s) resolve to Grade ≤1 or baseline value, with the following exceptions:

- Subjects may resume treatment in the presence of Grade 2 fatigue
- Subjects who have not experienced a Grade 3 drug-related skin AE may resume treatment in the presence of Grade 2 skin toxicity
- Drug-related pulmonary toxicity, diarrhea, or colitis, must have resolved to baseline before treatment is resumed
- Drug-related endocrinopathies adequately controlled with only physiologic hormone replacement may resume treatment

If the criteria to resume treatment are met, the subject should restart treatment at the next scheduled time point per protocol. However, if the treatment is delayed past the next scheduled time point per protocol, the next scheduled time point will be delayed until dosing resumes.

If treatment is delayed > 6 weeks, the subject must be permanently discontinued from study therapy, except as specified in discontinuation section.

**5.4 Management Algorithms:**

Immuno-oncology (I-O) agents are associated with AEs that can differ in severity and duration than AEs caused by other therapeutic classes. Nivolumab is considered an immuno-oncology agent in this protocol. Early recognition and management of AEs associated with immuno-oncology agents may mitigate severe toxicity. Management algorithms have been developed to assist investigators in assessing and managing the following groups of AEs:

Gastrointestinal, Renal, Pulmonary, Hepatic, Endocrinopathies, Skin, Neurological.

For subjects expected to require more than 4 weeks of corticosteroids or other immunosuppressants to manage an AE, consider recommendations provided in the algorithms. These algorithms are found in Appendix B of this protocol. The guidance provided in these algorithms should not replace the Investigator’s medical judgment but should complement it.

**5.5 Discontinuation Criteria:**

Treatment should be permanently discontinued for the following:

- Any Grade 2 drug-related uveitis or eye pain or blurred vision that does not respond to topical therapy and does not improve to Grade 1 severity within the re-treatment period OR requires systemic treatment
- Any Grade 3 non-skin, drug-related adverse event lasting > 7 days, with the following exceptions for drug-related laboratory abnormalities, uveitis, pneumonitis, bronchospasm, diarrhea, colitis, neurologic adverse event, hypersensitivity reactions, and infusion reactions:
  - Grade 3 drug-related uveitis, pneumonitis, bronchospasm, diarrhea, colitis, neurologic adverse event, hypersensitivity reaction, or infusion reaction **of any duration** requires discontinuation
  - Grade 3 drug-related laboratory abnormalities do not require treatment discontinuation except those noted below:
    - Grade 3 drug-related thrombocytopenia > 7 days or associated with bleeding requires discontinuation
    - Grade 3 drug-related ALT, AST or total bilirubin require discontinuation:
- Any Grade 4 drug-related adverse event or laboratory abnormality, except for the following events which do not require discontinuation:
  - Isolated Grade 4 amylase or lipase abnormalities that are not associated with symptoms or clinical manifestations of pancreatitis and decrease to < Grade 4 within 1 week of onset.
  - Isolated Grade 4 electrolyte imbalances/abnormalities that are not associated with clinical sequelae and are corrected with supplementation/appropriate management within 72 hours of their onset
- Any dosing interruption lasting > 6 weeks with the following exceptions:
- Dosing interruptions to allow for prolonged steroid tapers to manage drug-related adverse events are allowed. Prior to re-initiating treatment in a subject with a dosing interruption lasting > 6 weeks, the Investigator must be consulted. Tumor assessments should continue as per protocol even if dosing is interrupted
- Dosing interruptions > 6 weeks that occur for non-drug-related reasons may be allowed if approved by the Investigator. Prior to re-initiating treatment in a subject with a dosing interruption lasting > 6 weeks, the Investigator must be consulted. Tumor assessments should continue as per protocol even if dosing is interrupted
- Any adverse event, laboratory abnormality, or intercurrent illness which, in the judgment of the Investigator, presents a substantial clinical risk to the subject with continued nivolumab dosing

**5.6 Concomitant medications**

In general, the use of any concomitant medication/therapies deemed necessary for patient supportive care and safety are permitted. Other anticancer agents, specifically systemic chemotherapy, radiation therapy, or biologic response modifiers are not permitted during the study. Intrathecal chemotherapy is allowed if intended for prophylaxis. No other investigational drug is allowed during the study. Prophylaxis may be used for the prevention of infections or nausea and vomiting.

Subjects may be receiving systemic corticosteroids (daily doses ≤10 mg of prednisone or equivalent if indicated for adrenal replacement or antiemetic therapy), topical, or inhaled corticosteroids at study enrollment. They may receive systemic, topical, inhaled, or enteric corticosteroids while on study without limitation if they develop conditions that require corticosteroid therapy; such subjects are not required to discontinue study participation. The dose and amount of steroids that the patient receives will be recorded in the case report form.

All ongoing medications and therapies (including herbal products, nutritional supplements, and nontraditional medications) at screening will be considered prior medications. Concomitant medication data will not be collected or entered into the case report form other than steroids as mentioned above; however, the subject’s medication record will contain a list of concomitant medications.

6.0 PATIENT EVALUATION

6.1 Pre-Treatment Evaluation: All pretreatment studies should be obtained within 14 days (±3 days) of entry into the trial, unless otherwise stated.

1. A complete history and physical, including assessment of baseline adverse events.

2. CBC, platelet count, differential (differential can be omitted if WBC is ≤0.5 x10^9^/L).

3. Creatinine, total bilirubin, ALT, amylase, lipase.

4. Pregnancy test (urine or plasma) in females of childbearing potential should be performed within 24 hours of initiation of treatment.

5. Bone marrow aspirate during the last 14 days preceding study initiation. Cytogenetics will be obtained prior to therapy in patients known to have cytogenetic abnormalities at the time of diagnosis or relapse (results from prior analysis within 4 weeks can be used for this purpose).

6. Chest x-ray.

7. Pretreatment optional correlative studies will be collected on a companion protocol PA13-0291(Appendix C). Every effort will be made to collect all samples at all time-points for all patients; however, missing collection in one or more of these time points in occasional patients will not be considered a protocol deviation/violation.

8. Flow cytometry for MRD (peripheral blood and/or bone marrow) within 14 days.

9. Baseline TSH, Free T4 within 30 days of starting treatment.

6.2 Evaluation During Treatment: All tests are ±5 days unless otherwise specified.

1. Physical exam before administration of nivolumab for the first cycle, then every 4 weeks (±7 days) for 2 cycles, then every 8-12 weeks until end of 6 cycles of therapy. Patients who continue therapy beyond 6 months will continue having physical exam every 2-3 months while on therapy.

2. Assessment of adverse events every month (assessment may be by phone interview) while on therapy and approximately 30 days after end of therapy.

3. CBC, platelet count, differential (differential can be omitted if WBC is ≤0.5 x10^9^/L) once weekly for the first cycle, then every 2 weeks for two cycles, then every 4 weeks (±1 week).

4. Creatinine, total bilirubin, ALT once weekly for the first cycle, then every 2 weeks for two cycles, then every 4 weeks (±1 week). Amylase and lipase once per cycle.

5. TSH and Free T4 level every 3 months (±1 month) for the first 6 months (±1 month) and then every 6 months thereafter while on study.

6. Bone marrow aspiration on day 28 (± 7 days), then every 2 cycles (± 1 cycle) for 6 cycles, then every 3 months; then as clinically indicated. Bone marrow aspirations to be performed at MDACC.

7. Flow cytometry for MRD (peripheral blood and/or bone marrow) every 2 cycles (± 1 cycle) for 6 cycles, then every 3 months; then as clinically indicated.

8. Correlative studies (Appendix C). Every effort will be made to collect all samples at all-time points for all patients; however, missing collection in one or more of these time points in occasional patients will not be considered a protocol deviation/violation.

9. Patients who complete therapy will be followed as clinically indicated after the end of therapy. If treatment is resumed follow-up will be done according to the follow-up schedule that was being done at the time they discontinued therapy.

**6.3 End of Treatment Visit**

To be completed 30 days (+/- 7 days) after the last dose of study drug.

During this visit the following procedures will be performed:

Physical exam

Assessment of adverse events

CBC with differential and platelets

Creatinine, total bilirubin, ALT

Bone marrow aspiration (if not done within 6 weeks and if feasible)

Flow cytometry for MRD

Correlative studies (optional; missing optional studies collection will not be considered a protocol deviation)

If the patient is unwilling or unable to do end of study visit at MDACC, an assessment of adverse events may be done by phone and every attempt should be made to collect required studies from other institution if available.

7.0 CRITERIA FOR RESPONSE

The primary endpoint of this study is the rate of sustained remission at 6 months from the start of study treatment.

Response criteria will be modified from the International Working Group for AML (JCO 2003; 21: 4642-9). Responders are patients who obtain a CR, CRi, or PR, with or without cytogenetic response, hematologic improvements, and morphologic leukemia-free state. Briefly, criteria are as follows:

**Complete Remission (CR):**

- **Peripheral blood counts:**

Neutrophil count ≥1.0 x10^9^/L

Platelet count ≥100 x10^9^/L

- **Bone marrow aspirate and biopsy:**

≤5% blasts

No Auer rods

No extramedullary leukemia

**Complete remission with incomplete blood count recovery (CRi):**

- **Peripheral blood counts:**

Neutrophil count <1.0 x10^9^/L, or

Platelet count <100 x10^9^/L

- **Bone marrow aspirate and biopsy:**

< 5% blasts

No Auer rods

No extramedullary leukemia

**Partial Remission (PR)**:

- All CR criteria if abnormal before treatment except:
- ≥50 % reduction in bone marrow blast but still >5%

**Morphologic Leukemia-Free State (MLF)**:

- Bone marrow:
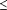
5% blasts

**Hematologic Improvement (HI):** Hematologic response must be described by the number of positively affected cell lines.

- **Erythroid response (E)** (pretreatment Hgb <11 g/dL):
  - Hgb increase by
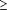
1.5 g/dL
- **Platelet response (P)** (pretreatment platelets <100 x10^9^/L):
  - Absolute increase of
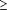
30 x 10^9^/L for patients starting with >20 x 10^9^/L platelets, or
  - Increase from < 20 x 10^9^/L to > 20 x 10^9^/L and by at least 100%
- **Neutrophil response (N)** (pretreatment ANC <1.0 x10^9^/L)
  - At least 100% increase and an absolute increase >0.5 x 10^9^/L
- **Blast response (B):**
  - At least 50% reduction in blast percentage.

**Relapse:** Morphologic relapse after CR is defined as a reappearance of leukemic blasts in the peripheral blood >2% or > 5% blasts in the bone marrow not attributable to any other cause (e.g. bone marrow regeneration after consolidation therapy). The appearance of new dysplastic changes should also be considered relapse. In the setting of recent treatment, if there are no or few (e.g., <2%) circulating blasts and the bone marrow contains 5% to 20% blasts, a repeat bone marrow performed at least a week later is necessary to distinguish relapse from bone marrow regeneration. In such instances the date of recurrence is defined as the first date that more than 5% blasts were observed in the marrow. The reappearance or development of morphologically proven extramedullary disease also indicates relapse. Molecular and/or genetic relapse is characterized by reappearance of a cytogenetic or molecular abnormality.

1. ADVERSE EVENT REPORTING
   1. Adverse event is any untoward medical occurrence that may present during treatment with a pharmaceutical product but which does not necessarily have a causal relationship with this treatment.

Adverse drug reaction is a response to a drug which is noxious and unintended and which occurs at doses normally used in man for prophylaxis, diagnosis, or therapy of disease or for the modification of physiologic function.

Assessing causal connections between agents and disease is fundamental to the understanding of adverse drug reactions. In general, a drug may be considered a contributory cause of an adverse event if, had the drug not been administered, 1) the event would not have happened at all, 2) the event would have occurred later than it actually did, or 3) the event would have been less severe.

The Investigator or physician designee is responsible for verifying and providing source documentation for all adverse events and assigning the attribution for each event for all subjects enrolled on the trial.

- 1. Adverse Events (AEs) will be evaluated according to the CTC AE version 4.0 and documented in medical record. Only unexpected AEs will be recorded in the Case Report Form (CRF). PDMS/CORe will be used as the electronic case report form for this protocol. Adverse events and protocol specific data will be entered into PDMS/CORe. Expected events during leukemia therapy are:
     1. Myelosuppression related events (due to disease or leukemia therapy)
        1. febrile or infection episodes not requiring management in the intensive care unit
        2. epistaxis or bleeding except for catastrophic CNS or pulmonary hemorrhage
        3. anemia, neutropenia, lymphopenia, thrombocytopenia, leukopenia
     2. **Disease Related Events**
        1. Symptoms associated with anemia
           1. fatigue
           2. weakness
           3. shortness of breath
        2. electrolyte abnormalities (sodium, potassium, bicarbonate, CO2, magnesium)
        3. chemistry abnormalities (LDH, phosphorus, calcium, BUN, protein, albumin, uric acid, alkaline phosphatase, glucose)
        4. coagulation abnormalities
        5. disease specific therapy (induction, maintenance, salvage, or stem cell therapy)
        6. alopecia
        7. bone, joint, or muscle pain
        8. liver function test abnormalities associated with infection or disease progression
        9. disease progression
        10. abnormal hematologic values
     3. **General Therapy Related Events**
        1. catheter related events
        2. renal failure related to tumor lysis syndrome or antibiotic/ antifungal therapy
        3. rash related to antibiotic use
     4. Hospitalization for the management of any of the above expected events (not considered SAE)
  2. Abnormal hematologic values will not be recorded on the case report form. For abnormal chemical values, the apogee or nadir (whichever is appropriate) will be reported per course on the case report form.
  3. Serious Adverse Event Reporting (SAE)

An adverse event or suspected adverse reaction is considered “serious” if, in the view of either the investigator or the sponsor, it results in any of the following outcomes:

- Death
- A life-threatening adverse drug experience – any adverse experience that places the patient, in the view of the initial reporter, at immediate risk of death from the adverse experience as it occurred. It does not include an adverse experience that, had it occurred in a more severe form, might have caused death.
- Inpatient hospitalization or prolongation of existing hospitalization
- A persistent or significant incapacity or substantial disruption of the ability to conduct normal life functions.
- A congenital anomaly/birth defect.
- Pregnancy
- Overdose of investigational agent
- Onset of a new secondary malignancy while on study.

Important medical events that may not result in death, be life-threatening, or require hospitalization may be considered a serious adverse drug experience when, based upon appropriate medical judgment, they may jeopardize the patient or subject and may require medical or surgical intervention to prevent one of the outcomes listed in this definition. Examples of such medical events include allergic bronchospasm requiring intensive treatment in an emergency room or at home, blood dyscrasias or convulsions that do not result in inpatient hospitalization, or the development of drug dependency or drug abuse (21 CFR 312.32).

- Important medical events as defined above, may also be considered serious adverse events. Any important medical event can and should be reported as an SAE if deemed appropriate by the Principal Investigator or the IND Sponsor, IND Office.
- All events occurring during the conduct of a protocol and meeting the definition of a SAE must be reported to the IRB in accordance with the timeframes and procedures outlined in “The University of Texas M. D. Anderson Cancer Center Institutional Review Board Policy for Investigators on Reporting Unanticipated Adverse Events for Drugs and Devices”. Unless stated otherwise in the protocol, all SAEs, expected or unexpected, must be reported to the IND Office, regardless of attribution (within 5 working days of knowledge of the event).
- **All life-threatening or fatal events,** that are unexpected, and related to the study drug, must have a written report submitted within **24** **hours** (next working day) of knowledge of the event to the Safety Project Manager in the IND Office.
- Unless otherwise noted, the electronic SAE application (eSAE) will be utilized for safety reporting to the IND Office and MDACC IRB.
- Serious adverse events will be captured from the time of the first protocol-specific intervention, until 30 days after the last dose of drug, unless the participant withdraws consent. Serious adverse events must be followed until clinical recovery is complete and laboratory tests have returned to baseline, progression of the event has stabilized, or there has been acceptable resolution of the event.
- Additionally, any serious adverse events that occur after the 30 day time period that are related to the study treatment must be reported to the IND Office. This may include the development of a secondary malignancy.

**Reporting to FDA:**

- Serious adverse events will be forwarded to FDA by the IND Sponsor (Safety Project Manager IND Office) according to 21 CFR 312.32.

**It is the responsibility of the PI and the research team to ensure serious adverse events are reported according to the Code of Federal Regulations, Good Clinical Practices, the protocol guidelines, the sponsor’s guidelines, and Institutional Review Board policy.**

**Investigator Communication with Supporting Company:**

Serious adverse event will be reported by FAX concurrently to Bristol-Myers Squibb (BMS) (Fax number: 609-818-3804) or BMS email: Worldwide.safety@bms.com. Information not available at the time of the initial report (e.g., an end date for the adverse event or laboratory values received after the report) must be documented on a follow-up report. A final report to document resolution of the SAE is required. Hospitalizations for the management of any expected adverse events (previously described) should be reported to BMS within 5 business days of the event.

**Pregnancies:** Any pregnancy that occurs during study participation or within 4 weeks of the patient’s last study drug administration should be reported to Bristol-Myers Squibb within 24 hours of learning of its occurrence.

The Investigator will follow the pregnant female until completion of the pregnancy. The Investigator will provide this information as a follow-up to the initial SAE.

If the outcome of the pregnancy meets the criteria for immediate classification as a SAE (i.e., spontaneous abortion [any congenital anomaly detected in an aborted fetus is to be documented], stillbirth, neonatal death, or congenital anomaly), the Investigator should follow the procedures for Expedited Reporting of SAEs.

**9.0 Statistical ConsiderationS**

This is a phase II study of Nivolumab as a maintenance therapy in patients with high risk AML and in first or second remission. The primary objective is to assess the recurrence-free survival rate at 6 months. It is expect the median RFS under the treatment is at least 8 months. Assume that RFS time follows an exponential distribution, the RFS rate at 6 months will be 59.5%. A maximum of 30 patients will be enrolled, and the study will monitor both futility and toxicity.

**Futility monitoring**

The study will be continuously monitoring RFS for futility, using the method of Thall, Wooten, and Tannir (2005). It is assumed that the RFS time is exponentially distributed with a median of λ_E_ among patients who receive nivolumab as a maintenance therapy, and a median of λ_H_ is assumed for the historical treatment. Further, we assume that λ_H_ follows an inverse gamma distribution, i.e., λ_H_ ~ IG(3.6, 20.8), which has a mean of 8 months and variance of 40. To reflect the little prior knowledge of λ_E_ we assume an inverse gamma prior distribution with the same mean of 8 months and a much larger variance of 500, i.e., λ_E_ ~ IG(2.128,9.024). The trial will be stopped early if Pr(λ_E_  > λ_H_ | data) < pL, where pL=0.25. That is, the study will be stopped if at any time during the study the data suggest that there is less than 25% probability that the median RFS was longer than that in the historical data. This futility monitoring rule will be first applied when 10 patients have been enrolled. A maximum of 30 patients will be enrolled into this study at an expected accrual rate of 2-3 patients per month. From historical experience, 25% of patients in this cohort may become eligible for stem cell transplant and may come off study. For purposes of this analysis, these patients will be censored at the time of receiving transplant. Patients will be followed up for an additional 6 months after completing treatment on protocol. Once these patients come off of study, they will be followed on a leukemia department long-term follow-up protocol and will not be receiving the evaluations described in section 6.2. The trial will be conducted using the Clinical Trial Conduct (CTC) website maintained by the Department of Biostatistics at MDACC. The website will be programmed with the operating characteristics of the Bayesian design of this protocol to allow continuous monitoring for futility.

The operating characteristics of the design, based on an overall assumed accrual rate of 2.5 patients per month and an additional follow-up of 6 months with 5000 simulated trials per scenario, are given in the following table. OneArmTTE (version 2.2) was used for the design and simulation.

| Scenario | True Median (months) | True RFS rate at 6 months | Pr(Stopped Early) | Mean No. patients (25%, 75%) | Average Trial Duration (months) |
| --- | --- | --- | --- | --- | --- |
| 1 | 2 | 12.50% | 1 | 10.5 (10, 10) | 4.24 |
| 2 | 4 | 35.40% | 0.86 | 16.7 (10, 23) | 7.83 |
| 3 | 6 | 50% | 0.36 | 24.1 (14, 30) | 13.56 |
| 4 | 8 | 59.50% | 0.16 | 27.2 (30, 30) | 16.01 |
| 5 | 10 | 66% | 0.08 | 28.6 (30, 30) | 16.98 |
| 6 | 12 | 70.70% | 0.04 | 29.2 (30, 30) | 17.4 |

**Toxicity monitoring**

In addition, we will continuously monitor treatment-related toxicities using the Bayesian approach of Thall, Simon, Estey (1995). Specifically, the trial will be stopped if Prob(π_T_>0.30 | data)>0.88, where π_T_ is the toxicity rate and we assume a beta (0.6, 1.4) prior for πT That is, if at any given time, there is more than 88% probability that the toxicity rate of the maintenance therapy with nivolumab is greater than 30%, the trial will be stopped. For the purpose of toxicity monitoring, toxicities are defined as any treatment –related clinically significant >/= grade 3 non-hematologic AEs occurred any time during the trial. We will apply this stopping rule starting from the 10^th^ patient. The stopping boundaries corresponding to this toxicity monitoring rule are described in the following table:

**Stopping boundaries for treatment-related grade 3, 4 non-hematologic toxicities**

| The number of patients evaluated for toxicities | 10 -12 | 13-15 | 16-18 | 19-20 | 21-23 | 24-26 | 27-29 |
| --- | --- | --- | --- | --- | --- | --- | --- |
| Stop the trial if greater than or equal to this many of patients with toxicities | 6 | 7 | 8 | 9 | 10 | 11 | 12 |

The operating characteristics for toxicity monitoring are described in the following table. MultcLean Desktop 2.1.0 was used for the design and simulations for toxicity monitoring.

**Operating characteristics for Toxicity Monitoring**

| True Toxicity Rate | Early Stopping Probability | Sample Size | | |
| --- | --- | --- | --- | --- |
|  |  | 25^th^ percentile | Median | 75^th^ percentile |
| 0.1 | 0.0007 | 30 | 30 | 30 |
| 0.2 | 0.0353 | 30 | 30 | 30 |
| 0.3 | 0.2447 | 29 | 30 | 30 |
| 0.4 | 0.635 | 11 | 18 | 30 |
| 0.5 | 0.9134 | 10 | 11 | 15 |

A toxicity summary will be submitted to the IND medical monitor after every 3 patients starting from the 10^th^ patient.

For the primary analysis of the efficacy endpoint, we will use Kaplan-Meier methodology to compute the RFS rate at 6 months and the 95% confidence interval. As a secondary analysis, we will perform a competing risk analysis treating stem cell transplant as a competing event for RFS.” The Kaplan-Meier method will be used to estimate the median RFS time and the probability of overall survival. Expressions of immune mediated markers will be summarized with descriptive statistics: means medians, ranges, and standard deviations. Two-sample t test or Wilcoxon rank sum test will be used to compare the expressions of markers between patients remaining in CR and patients having recurrence disease. Scatterplots will be used to explore possible associations between clinical responses and the immunomodulatory markers. Spearman correlation coefficients will be computed to evaluate the association between MRD and immunomodulatory markers, as well as the association between MRD and the changes in marker expressions pre and post treatment.

# 1.6 Supplementary References

1. Robinson JT, Thorvaldsdóttir H, Winckler W, et al. Integrative genomics viewer. *Nat Biotechnol*. 2011;29(1):24-26.

2. Warren M, Luthra R, Yin CC, et al. Clinical impact of change of FLT3 mutation status in acute myeloid leukemia patients. *Mod Pathol*. 2012;25(10):1405-1412.

3. Jaso JM, Wang SA, Jorgensen JL, Lin P. Multi-color flow cytometric immunophenotyping for detection of minimal residual disease in AML: past, present and future. *Bone Marrow Transplant*. 2014;49(9):1129-1138.

4. Shah MV, Jorgensen JL, Saliba RM, et al. Early Post-Transplant Minimal Residual Disease Assessment Improves Risk Stratification in Acute Myeloid Leukemia. *Biol Blood Marrow Transplant*. 2018;24(7):1514-1520.

5. Molldrem J, Dermime S, Parker K, et al. Targeted T-cell therapy for human leukemia: cytotoxic T lymphocytes specific for a peptide derived from proteinase 3 preferentially lyse human myeloid leukemia cells. *Blood*. 1996;88(7):2450-2457.

6. Dengler R, Munstermann U, al-Batran S, et al. Immunocytochemical and flow cytometric detection of proteinase 3 (myeloblastin) in normal and leukaemic myeloid cells. *Br J Haematol*. 1995;89(2):250-257.

7. Molldrem JJ, Clave E, Jiang YZ, et al. Cytotoxic T lymphocytes specific for a nonpolymorphic proteinase 3 peptide preferentially inhibit chronic myeloid leukemia colony-forming units. *Blood*. 1997;90(7):2529-2534.

8. Qazilbash MH, Wieder E, Rios R, et al. Vaccination with the PR1 Leukemia-Associated Antigen Can Induce Complete Remission in Patients with Myeloid Leukemia. *Blood*. 2004;104:77a (Abstract #259).

9. Scheibenbogen C, Letsch A, Thiel E, et al. CD8 T-cell responses to Wilms tumor gene product WT1 and proteinase 3 in patients with acute myeloid leukemia. *Blood*. 2002;100(6):2132-2137.

10. Pizzolo G, Trentin L, Vinante F, et al. Natural killer cell function and lymphoid subpopulations in acute non-lymphoblastic leukaemia in complete remission. *Br J Cancer*. 1988;58(3):368-372.

11. Archimbaud E, Bailly M, Dore JF. Inducibility of lymphokine activated killer (LAK) cells in patients with acute myelogenous leukaemia in complete remission and its clinical relevance. *Br J Haematol*. 1991;77(3):328-334.

12. Lauria F, Raspadori D, Rondelli D, Ventura MA, Foa R. In vitro susceptibility of acute leukemia cells to the cytotoxic activity of allogeneic and autologous lymphokine activated killer (LAK) effectors: correlation with the rate and duration of complete remission and with survival. *Leukemia*. 1994;8(5):724-728.

13. Lowdell MW, Koh MB. Immunotherapy of AML: future directions. *J Clin Pathol*. 2000;53(1):49-54.

14. Cortes JE, Kantarjian HM, O'Brien S, et al. A pilot study of interleukin-2 for adult patients with acute myelogenous leukemia in first complete remission. *Cancer*. 1999;85(7):1506-1513.

15. Brune M, Castaigne S, Catalano J, et al. Improved leukemia-free survival after postconsolidation immunotherapy with histamine dihydrochloride and interleukin-2 in acute myeloid leukemia: results of a randomized phase 3 trial. *Blood*. 2006;108(1):88-96.

16. Sakuishi K, Apetoh L, Sullivan JM, Blazar BR, Kuchroo VK, Anderson AC. Targeting Tim-3 and PD-1 pathways to reverse T cell exhaustion and restore anti-tumor immunity. *J Exp Med*. 2010;207(10):2187-2194.

17. Zhou Q, Munger ME, Veenstra RG, et al. Coexpression of Tim-3 and PD-1 identifies a CD8+ T-cell exhaustion phenotype in mice with disseminated acute myelogenous leukemia. *Blood*. 2011;117(17):4501-4510.

18. Iwai Y, Terawaki S, Honjo T. PD-1 blockade inhibits hematogenous spread of poorly immunogenic tumor cells by enhanced recruitment of effector T cells. *Int Immunol*. 2005;17(2):133-144.

19. Hirano F, Kaneko K, Tamura H, et al. Blockade of B7-H1 and PD-1 by monoclonal antibodies potentiates cancer therapeutic immunity. *Cancer Res*. 2005;65(3):1089-1096.

20. Hamid O, Robert C, Daud A, et al. Safety and tumor responses with lambrolizumab (anti-PD-1) in melanoma. *N Engl J Med*. 2013;369(2):134-144.

21. Weber JS, Kudchadkar RR, Yu B, et al. Safety, efficacy, and biomarkers of nivolumab with vaccine in ipilimumab-refractory or -naive melanoma. *J Clin Oncol*. 2013;31(34):4311-4318.

22. Keir ME, Butte MJ, Freeman GJ, Sharpe AH. PD-1 and its ligands in tolerance and immunity. *Annu Rev Immunol*. 2008;26:677-704.

23. Nishimura H, Okazaki T, Tanaka Y, et al. Autoimmune dilated cardiomyopathy in PD-1 receptor-deficient mice. *Science*. 2001;291(5502):319-322.

24. Nishimura H, Nose M, Hiai H, Minato N, Honjo T. Development of lupus-like autoimmune diseases by disruption of the PD-1 gene encoding an ITIM motif-carrying immunoreceptor. *Immunity*. 1999;11(2):141-151.

25. Nomi T, Sho M, Akahori T, et al. Clinical significance and therapeutic potential of the programmed death-1 ligand/programmed death-1 pathway in human pancreatic cancer. *Clin Cancer Res*. 2007;13(7):2151-2157.

26. Iwai Y, Ishida M, Tanaka Y, Okazaki T, Honjo T, Minato N. Involvement of PD-L1 on tumor cells in the escape from host immune system and tumor immunotherapy by PD-L1 blockade. *Proc Natl Acad Sci U S A*. 2002;99(19):12293-12297.

27. Topalian SL, Hodi FS, Brahmer JR, et al. Safety, activity, and immune correlates of anti-PD-1 antibody in cancer. *N Engl J Med*. 2012;366(26):2443-2454.
